# Clinical and Immunological Markers of Dengue Progression in a Study Cohort from a Hyperendemic Area in Malaysia

**DOI:** 10.1371/journal.pone.0092021

**Published:** 2014-03-19

**Authors:** Anusyah Rathakrishnan, Benjamin Klekamp, Seok Mui Wang, Thamil Vaani Komarasamy, Santha Kumari Natkunam, Jameela Sathar, Azliyati Azizan, Aurora Sanchez-Anguiano, Rishya Manikam, Shamala Devi Sekaran

**Affiliations:** 1 Department of Medical Microbiology, Faculty of Medicine, University of Malaya, Kuala Lumpur, Malaysia; 2 Department of Global Health, College of Public Health, University of South Florida, Tampa, Florida, United States of America; 3 Institute of Medical Molecular Biotechnology, Faculty of Medicine, University Technology Mara, Selangor, Malaysia; 4 Hospital Tengku Ampuan Rahimah, Persiaran Tengku Ampuan Rahimah, Klang, Selangor; 5 Clinical Hematology Laboratory, Department of Hematology, Hospital Ampang, Ampang, Selangor, Malaysia; 6 Department of Trauma and Emergency Medicine, University Malaya Medical Centre, Kuala Lumpur, Malaysia; Tulane University, United States of America

## Abstract

**Background:**

With its elusive pathogenesis, dengue imposes serious healthcare, economic and social burden on endemic countries. This study describes the clinical and immunological parameters of a dengue cohort in a Malaysian city, the first according to the WHO 2009 dengue classification.

**Methodology and Findings:**

This longitudinal descriptive study was conducted in two Malaysian hospitals where patients aged 14 and above with clinical symptoms suggestive of dengue were recruited with informed consent. Among the 504 participants, 9.3% were classified as non-dengue, 12.7% without warning signs, 77.0% with warning signs and 1.0% with severe dengue based on clinical diagnosis. Of these, 37% were misdiagnosed as non-dengue, highlighting the importance of both clinical diagnosis and laboratory findings. Thrombocytopenia, prolonged clotting time, liver enzymes, ALT and AST served as good markers for dengue progression but could not distinguish between patients with and without warning signs. HLA-A*24 and -B*57 were positively associated with Chinese and Indians patients with warning signs, respectively, whereas A*03 may be protective in the Malays. HLA-A*33 was also positively associated in patients with warning signs when compared to those without. Dengue NS1, NS2A, NS4A and NS4B were found to be important T cell epitopes; however with no apparent difference between with and without warning signs patients. Distinction between the 2 groups of patients was also not observed in any of the cytokines analyzed; nevertheless, 12 were significantly differentially expressed at the different phases of illness.

**Conclusion:**

The new dengue classification system has allowed more specific detection of dengue patients, however, none of the clinical parameters allowed distinction of patients with and without warning signs. While the HLA-A*33 may be predictive marker for development of warning signs; larger studies will be needed to support this findings.

## Introduction

Malaysia is a multiracial country with an estimated population of 28 million people [1]. Over the years, the country has achieved tremendous improvement in its health sector; however, infectious diseases remain as major causes of high morbidity and mortality rates. Amongst the communicable diseases, dengue has the highest incidence rates (167.8/100000 population) in Malaysia [1]. It is also widespread throughout the tropical and subtropical regions of the world, with an estimated two-fifth of the world population being at risk of infection [2].

Previously classified as dengue fever (DF), dengue hemorrhagic fever (DHF) and dengue shock syndrome (DSS), currently according to the WHO dengue classification 2009, dengue is classified as with or without warning signs and severe dengue [3]. Usually, the initial clinical symptoms of dengue patients will not be able to differentiate mild dengue from severe cases. As the disease progresses, patients are classified via their clinical presentation over time and also via laboratory confirmation. Generally, a dengue virus (DENV) infected person may be asymptomatic or may just develop undifferentiated fever, typically with rashes, body aches and pains, nausea, vomiting and diarrhea. This patient may then recover or may further deteriorate and develop warning signs which include persistent vomiting, abdominal pain and tenderness, bleeding tendencies, fluid accumulation, hepatomegaly, with increased hematocrits and decreased platelets. In this critical phase, if not clinically well-managed, severe plasma leakage, bleeding and organ impairment may occur and can be fatal. Currently, patients with warning signs require admission into healthcare facilities for in-hospital management and care [3]. Since Malaysia is dengue hyperendemic [4], the social and economic burden as well as the disability-adjusted life years (DALYs) of the country is on a continuous rise [5]. In 2010, Malaysia had 46171 cases of reported dengue with 134 deaths, unreported and misdiagnosed cases unaccounted for.

The complexity in dengue pathogenesis has hampered the development of vaccines and antiviral drugs. Despite decades of research efforts, dengue immunopathogenesis has become more complex as various contradicting and controversial findings are being uncovered around the world [6]. Antibody enhancement [7], improper T cell [8], [9] and cytokine response [10], [11], and host genetic factors [12] are amongst the postulated immunopathogenesis leading to severe dengue.

Here, we report the clinical epidemiology of dengue disease in patients from Klang Valley, Malaysia from 2 major hospitals in the region. We assessed the clinical and immunological profiles of dengue suspected patients in hopes of identifying clinical/biomarkers to enable early diagnosis of disease with respect to the WHO 2009 classification.

## Methodology

### Study Design and Study Population

This longitudinal descriptive study was conducted in the emergency department and dengue wards of two hospitals situated in the Klang Valley, Malaysia: Ampang Hospital (Ampang, Selangor) and Tengku Ampuan Rahimah Hospital (Klang, Selangor) from June 2010 to April 2011. Study inclusion criteria included: (i) patients above the age of 18 or if between 14–18 years of age with parental consent and (ii) with clinical symptoms suggestive of dengue as described in the WHO 2009 dengue classification and determined by medical officers. Patients who met the criteria were recruited with written informed consent was obtained. For minors written parental consent was obtained on behalf of the 14–18 year-old group. Ethical clearances were obtained for all procedures performed from all hospitals and laboratory involved: University Malaya Medical Center (782.90), Ampang Hospital (NMRR-10-683-6420), Tengku Ampuan Rahimah (NMRR-10-683-6420) and University of South Florida (Pro00000425). The study was conducted in keeping with the Declaration of Helsinki (amended Seoul, Korea 2008) on human studies.

### Clinical Data Collection

A standardized form for clinical data collection was designed and used throughout the recruitment process. The clinical data collected for this study include (i) demographics, (ii) co-morbidities, (iii) hospital laboratory findings (iv) clinical symptoms (v) blood, liver and kidney profiles at three different phases of illness and (vi) clinical diagnosis. The classification of dengue patients was determined by clinicians based on the criteria of the WHO 2009 dengue classification using clinical diagnosis and also the hospital laboratory diagnostics. The 2009 dengue classification classifies dengue patients into non-dengue (ND), dengue without warning signs (DwoWS), dengue with warning signs (DwWS) and severe dengue (SD). Curve fitting was done using the LOWESS coarse curve to follow the general trends of clinical parameters in the DwoWS and DwWS patients by the day of illness (DOI). Statistical analysis was performed to compare the differences in clinical parameters between patient groups using the two-tailed Kruskal-Wallis one-way analysis of variances (ANOVA), followed by Dunn’s multiple comparison test. All analyses were done via GraphPad Prism 5 for Windows version 5.01.

### Blood Collection and Processing

Venous blood was collected from each participant at three different disease phases: febrile, defervescence and convalescence. Febrile phase was when the patient presented typical clinical symptoms of dengue including fever, rash, and body aches. Defervescence was when patient’s fever had abated and an increase was noted in capillary permeability or hematocrit with concurrent decrease in platelets, as well as manifestations of various signs of severity lasting for 24–48 hours. Lastly the convalescence phase samples were collected a week after the defervescence stage; however with certain exceptions in which patients could not follow up at the appointed time. The blood collected was separated for serum, plasma as well as PBMC and were stored at −80°C until further use.

### Independent Laboratory Diagnosis of Dengue

As both hospitals conduct only serological diagnostics for dengue including IgM detection and in some cases, NS1 detection, a separate and independent laboratory diagnostic testing was performed to confirm the infection status. Serum samples collected at the 3 different phases from all patients were subjected to several diagnostics assays. Dengue viral RNA was detected using one-step SYBR green I real-time RT-qPCR [13]. Dengue specific IgM was detected using an in-house capture IgM ELISA [14], whereas dengue total antibodies was determined via haemagglutination inhibition (HI) assay [15]. Dengue NS1 was detected using the Pan-E Dengue Early ELISA kit (Panbio, Queensland, Australia). Nevertheless, the findings from this independent testing were not incorporated with the hospitals’ findings and were not taken into consideration during patient classification.

### DNA Extraction and HLA Typing

Genomic DNA of dengue patients were extracted from whole blood via the AccuPrep Genomic DNA Extraction kit (Bioneer, Korea). The extracted DNA was then used in HLA typing via the Olerup SSP HLA Typing Kits without *Taq* polymerase. For determination of HLA-A and HLA-B alleles, the HLA-A Low Resolution Kits (Lot No. 62G, 04L, 02N) and HLA-B Low Resolution Kits (Lot No. 20K, 06L, 03N) were applied, respectively. Interpretations of the HLA alleles were then done with (i) lot-specific interpretation and specificity tables provided by the manufacturer and (ii) SCORE program designed based on “Virtual DNA Analysis” (Helmberg SCORE, Olerup SSP). The association between allele prevalence and dengue infection was examined using Microsoft Excel and GraphPad Prism 5 for Windows version 5.01. For each HLA allele, the proportion of DENV infected patients and control subjects with the allele were compared, using allele frequency (AF) values with the formula [16], AF = No of occurrences of a particular allele divided by 2n, where n is the number of individuals studied. The degree of association between HLA alleles and disease state was then expressed as the odds ratio (OR), which was obtained from standard contingency table analysis. Groups with higher OR value suggest increased risk of infection, and vice versa. The P value was determined by using the two-tailed Fisher’s exact test with p<0.05 being significant. Due to the small number of patients in the ND and SD classifications, the clinically diagnosed ND and DwoWS were grouped into one category, without warning signs; whereas the DwWS and SD patients were categorized into with warning signs.

### PBMC Isolation and IFN-γ Enzyme Linked ImmunoSpot (ELISpot) Assay

Thirty-six peptides with lengths of 9–10mers were designed based on Malaysian circulating DENV strains, using two major histocompatibility complex ligands and peptide motifs databases; SYFPEITHI [17] or RANKPEP [18] (Table S1). The peptides were synthesized with more than 90% purity (JPT Peptide Technologies GmbH, Germany) and were then used in the IFN-γ Elispot assay to determine the T cell responses in dengue patients, where activated IFN-γ will be released and quantitated in a standard direct ELISA format on nitrocellulose plates [9]. The spots were read and counted with Zeiss KS Elispot reader (Carl Zeiss, Germany). The number of spots per well were expressed as spot forming cells (SFCs) per million PBMCs. A response was considered significant when the number of SFCs for the patients was at least twice of the negative control and with a minimum of 50 SFCs per million. As above, the clinically diagnosed ND and DwoWS were grouped into one category, without warning signs; whereas the DwWS and SD patients were categorized into with warning signs. The two-tailed unpaired *t*-test with Welch’s correction was used to compare between the groups without and with warning signs. To compare between different peptide pools and individual peptides, one-way ANOVA with Kruskal-Wallis test, followed by Dunn’s Multiple Comparison test was applied. A probability of less than 0.05 was considered as significant.

### Identification of Cytokines, Chemokines and Growth Factors

The levels of 16 cytokines (IL-1β, IL-1ra, IL-2, IL-4, IL-5, IL-6, IL-9, IL-10, IL-12, IL-13, IL-15, IL-17, IFN-γ, MIF, TNF-α, and TNF-β), 8 chemokines (CCL2, CCL3, CCL4, CCL5, CCL11, CXCL10, CXCL12 and IL-8), 7 growth factors (FGF-2, G-CSF, GM-CSF, HGF, IL-7, PDGF and VEGF) and 2 adhesion molecules (ICAM-1 and VCAM-1) in dengue patients’ sera were evaluated via BioPlex Pro Assays (Bio-Plex Human Cytokine Assay; Bio-Rad Inc., Hercules, CA, USA) [19]. The fluorescent signals were read using the Bio-Plex 200 System (Bio-Rad Laboratories). Via the Bio-Plex Manager 6.0 Software, raw data was measured as the relative fluorescence intensity and then converted to cytokines concentration based on the standard curve generated (Bio-Rad Laboratories). Once again, the ND and DWoWS patients were grouped as “without warning signs” and the DwWS and SD patients were grouped as “with warning signs”. The differences in cytokine levels between the differences groups dengue patients were evaluated using the Kruskal-Wallis one way analysis of variance (ANOVA), followed by Dunn’s multiple comparison test. All statistical analyses performed were done using GraphPad Prism 5 for Windows, Version 5.01 (San Diego, California, USA).

## Results

### Characteristics of Study Population- the Klang Valley, Malaysia Scenario

The study was conducted in two government-based hospitals located in the Klang Valley; Ampang Hospital and Tengku Ampuan Rahimah Hospital Klang. A total of 512 patients who fit the study criteria were recruited at both hospitals and of these, 8 withdrew. The study participants whom had an average age 29.5 years and were mostly male (75.6%) were mainly recruited in the dengue wards of both hospitals (83.7%). As a multinational country, the study group when segregated ethnically had 65.5% of Malays, 13.5% Chinese, 11.7% Indians and 9.3% of other ethnicities. According to the new WHO classification, 9.3% were classified as ND, 12.7% as DwoWS, 77.0% as DwWS and 1.0% as SD (Table 1).

**Table 1:** Demographics on study population based on clinical diagnosis.

	ND	DwoWS	DwWS	SD
**No. Patients**	47	64	388	5
**Diagnosed with hepatitis** a	3	16	165	2
**Recruitment area**				
Emergency	24	10	46	2
Ward	23	54	342	3
**Age (Mean ± SEM)**	15–81 (29.1±2.0)	13–60 (29.9±1.5)	12–77 (29.5±0.6)	22–39 (27.5±2.6)
**Sex**				
Female	13	11	98	1
Male	34	53	290	4
**Race**				
Malay	31	37	259	3
Chinese	3	11	52	2
Indian	8	7	44	0
Others	5	9	33	0
**Pregnancy**	1	0	2	1
**Previous DENV Infection** b	1	2	22	0

ND: Non dengue; DwoWS: Dengue without warning signs; DwWS: Dengue with warning signs; SD: Severe dengue; SEM: Standard error mean;

a Discharged with the respective classification and with hepatitis;

b Previous known dengue infections to the knowledge of the patient.

**Figure 1:**
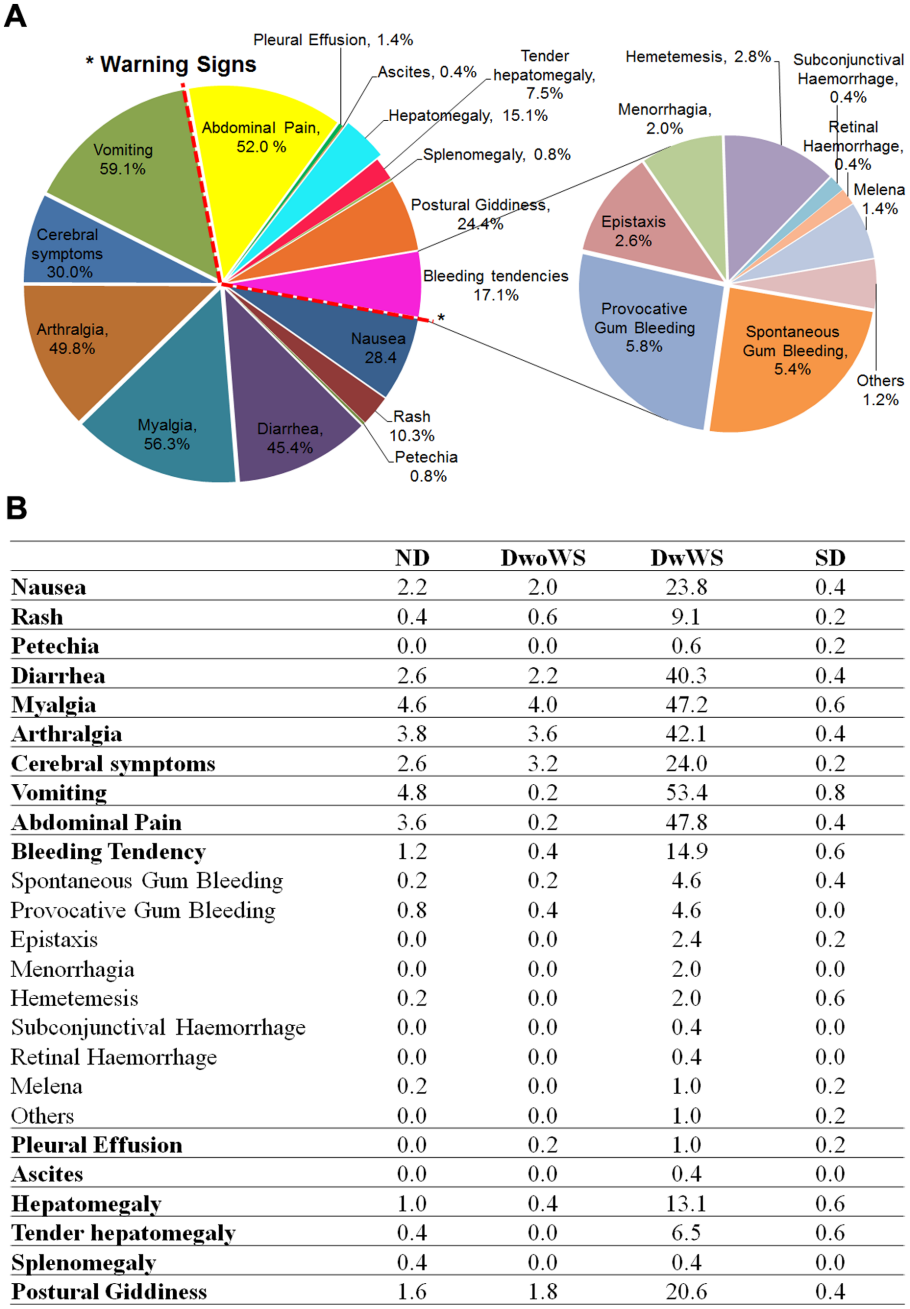
Clinical symptoms of the study population. *indicates the symptoms/manifestations considered as warning signs by the WHO 2009 dengue classification. Detailed clinical symptoms (in percentages) by dengue classification are given in the table.

Generally, participants sought medical intervention during the 5^th^ DOI, with the admission average also at 5^th^ DOI. The clinical symptoms of the dengue suspected patients in general included vomiting (59.1%), myalgia (56.3%), arthralgia (49.8%), diarrhea (45.4%), various cerebral symptoms including headaches, and retro-orbital pain (30.0%) nausea (28.4%), rashes (10.3%) and petechia (0.8%) (Figure 1). The warning signs in patients included a concurrent increase in hematocrit with the decrease of platelets, abdominal pain and tenderness (52.0%), bleeding tendencies (17.1%), postural giddiness (24.4%), hepatomegaly and tenderness (22.6%), splenomegaly (0.8%), pleural effusion (1.4%) and ascites (0.4%). An overwhelming 42.9% of recruited patients were also diagnosed with acute hepatitis (Table 1).

In both hospitals, the commonly ordered diagnostics assay is the IgM test, while only 26% of the study cohort was tested for dengue NS1 (Table 2). From the hospital diagnostics, 44.6% of patients were IgM positive and 10.3% were NS1 positive. However, as the clinical diagnosis was based solely on clinical symptoms and serological data, we applied various other diagnostic assays to confirm the true status of the recruited cohort before embarking on further investigations. However, we would like to note that these laboratory assays were not included in the classification of dengue patients. From the 4 separate diagnostic assays conducted, a patient was considered dengue positive when (i) dengue virus and/or antigen was detected, or (ii) dengue IgM seroconversion occurred in paired sera, or (iii) dengue total antibodies had a fourfold rise in titers in paired sera and (iv) a combination of the above. A presumptive dengue patient, on the other hand, had either IgM of HI titer of above 1280 but without seroconversion or fourfold rise, respectively, occurring mainly in patients with single serum samples. Six patients were not included in the diagnostic assays, as their blood was in unbefitting conditions upon receipt. From the laboratory tests, we found 311 patients to be positive for current dengue infection, 54 with presumptive dengue and 133 negative for all tests carried out (Table 3). Dengue virus was detected in 49 patients with prevailing serotypes of DENV1 (69.4%) and DENV3 (30.6%). Dengue NS1 was detected in 218 patients, a number much higher than detection of the virus itself. Out of the 322 patient who had dengue IgM, 15.2% had seroconversion in their defervescence/convalescence samples. Via the HI assay, we determined that 221 patients had primary dengue infections and 140 had presumptive/confirmed secondary infections, whereas 4 could not be determined.

**Table 2:** Hospital laboratory diagnostics specifically Dengue IgM and Dengue NS1.

	ND			DwoWS			DwWS			SD		
	−ve	+ve	ND	−ve	+ve	ND	−ve	+ve	ND	−ve	+ve	ND
IgM^a^	19	6	22	24	29	11	109	188	91	3	2	0
NS1^b^	5	2	40	16	12	36	59	38	291	0	0	5

ND: Non dengue; DwoWS: Dengue without warning signs; DwWS: Dengue with warning signs; SD: Severe dengue; IgM: Immunoglobulin M; NS1: Non-structural protein 1; −ve: negative; +ve: positive; ND: not determined;

a IgM ELISA done in the hospital laboratory;

b NS1 ELISA done in the hospital laboratory.

**Table 3:** Laboratory diagnostics confirmation of study population.

Laboratory Diagnostics	ND	DwoWS	DwWS	SD
**Positive**	**12**	**37**	**259**	**3**
IgM with seroconversion	0	1	4	0
NS1	3	2	12	0
HI	0	0	4	0
IgM+NS1	2	13	77	1
IgM+HI	2	7	68	2
IgM+RT-PCR	0	0	1	0
RT-PCR+NS1	1	2	5	0
NS1+HI	0	1	3	0
IgM+RT-PCR+NS1	1	2	15	0
IgM+RT-PCR+HI	0	0	4	0
IgM+NS1+HI	1	4	55	0
RT-PCR+NS1+ HI	0	0	1	0
IgM+NS1+HI+RT-PCR	2	5	10	0
**Presumptive**	**5**	**6**	**43**	**0**
IgM ONLY	4	6	35	0
HI >1280 in a single serum	1	0	8	0
**Negative**	**29**	**21**	**81**	**2**

ND: Non dengue; DwoWS: Dengue without warning signs; DwWS: Dengue with warning signs; SD: Severe dengue; IgM: Immunoglobulin M; NS1: Non-structural protein 1; HI: Hemagglutination Inhibiton Assay; +: combination.

The rate co-morbidities in this study group were relatively low (Table 4), where 4.2% of patients had diabetes mellitus, 3.6% had hypertension, 1.4% had ischemic heart disease, 0.6% had congestive heart failure and 0.2% had chronic kidney disease. Patients in this study required an average of 4 (SEM ±0.4) days of admission, however there were about 2.2% who had been admitted for more than 10 days. On average, the febrile blood draw was around day 5 of illness (SEM: ±0.3), the defervescence draw was at day 6 of illness (SEM: ±0.3) and the convalescence draw was at day 11 of illness (SEM: ±0.7).

**Table 4:** Co-morbidities in the study population.

	ND	DwoWS	DwWS	SD
**Diabetes mellitus**	4	3	14	0
**Hypertension**	1	3	14	0
**Chronic kidney disease**	0	0	1	0
**Ischemic heast disease**	0	1	6	0
**Congestive heart failure**	0	1	2	0

ND: Non dengue; DwoWS: Dengue without warning signs; DwWS: Dengue with warning signs; SD: Severe dengue.

**Figure 2:**
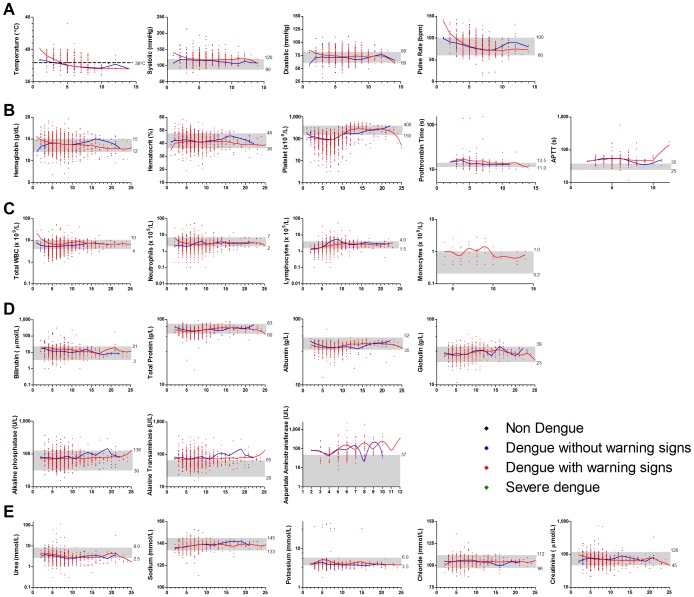
Clinical profile of study population. Scatter plot of the study population’s (A) vital signs (temperature, systolic, diastolic and pulse rate); (B) blood profile (hemoglobin, hematocrit, platelet, prothrombin time, activated partial thromboplastin time); (C) white blood cell profile (total white blood cells, lymphocytes, neutrophils and monocytes); (D) liver profile (total protein, total billirubin, albumin, globulin, alkaline phosphatase, alanine transaminase, aspartate aminotransferase) and (E) kidney profile (urea, sodium, potassium, chloride and creatinine) over days of illness. The LOWESS curves follows the general trend of the observed clinical parameter in dengue patients without (blue) and with (red) warning signs. Grey zone shows the reference range values.

Analyses of the patients’ vital signs (Figure 2A) and kidney profile (Figure 2E) revealed no significant differences between the classifications and from the normal range. The mean hematocrit levels were increased only in the DwoWS patients (53.1±9.8%), and these were significantly different from the ND group at febrile (p = 0.0051) and convalescence (p = 0.0052). The average platelet counts were low in all four groups during febrile and defervescence. Significant differences were observed between ND group with DwoWS group during febrile (p = 0.0285) and with DwWS during defervescence (p<0.0001). Both the prothrombin time (PT) and activated partial thromboplastin time (APTT) were higher in both DwoWS and DwWS groups. Generally, the white blood cells, neutrophils and monocytes levels were in the reference range (Figure 2C). However, a slight decrease was noted in the lymphocyte count of DwoWS (1.1±0.1×10^9^/L), DwWS (1.2±0.1×10^9^/L) and SD (0.65±0.3×10^9^/L) patients at the febrile phase. The total protein, bilirubin, albumin, globulin and alkaline phosphatase levels in all groups were normal (Figure 2D). However, a significant difference (p = 0.0015) in total bilirubin levels was noted between DwoWS (13.2±1.8 μmol/L) and DwWS (9.4±0.4 μmol/L) at febrile phase. A considerable dissimilarity (p = 0.0056) was also noted in the levels of total protein between DwoWS (66.3±1.1 g/L) and DwWS (62.3±0.5 g/L) at defervescence. Alanine transaminase (ALT) was found to be in higher levels in all four dengue classified groups while aspartate aminotransferase (AST) was only found to be higher in DwoWS and DwWS patients throughout the illness.

### MHC Class I- HLA-A and -B Alleles Distribution and Association with Dengue Infection

**Figure 3:**
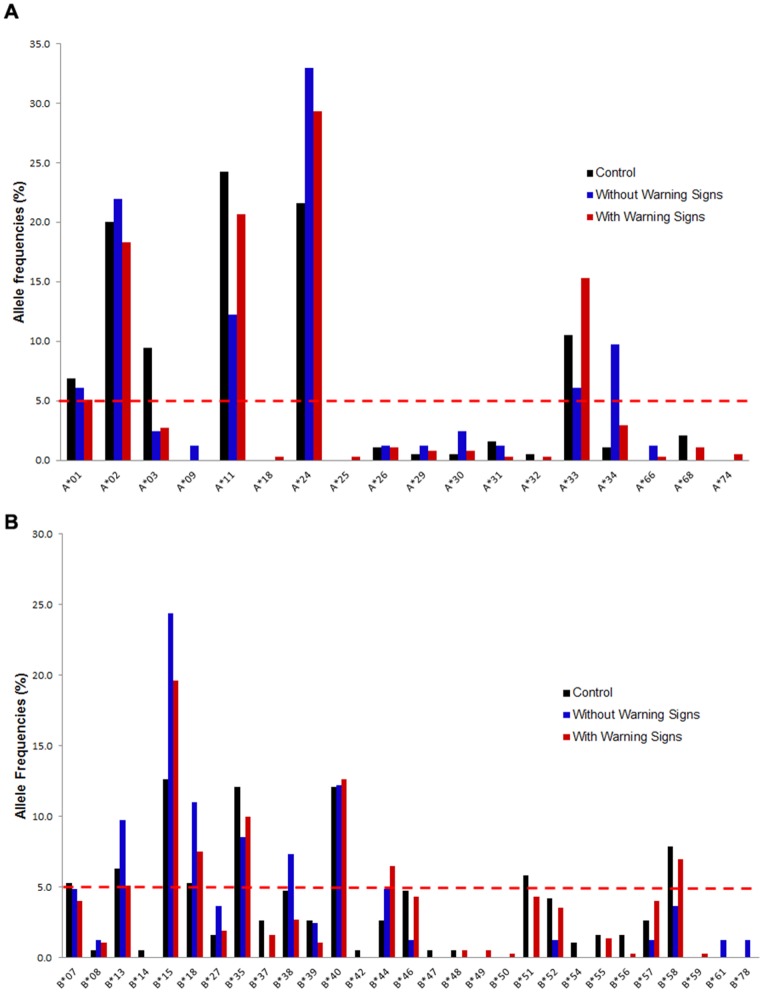
HLA Allele Frequencies in the Study Population. (A) HLA-A alleles in the total study population; (B) HLA-B alleles in the total study population.

The adaptive immune system plays an important role in host defense against viral infections and a major component of this system is the MHC molecules, responsible for antigen display. Pathogenic variants of MHC molecules have been hypothesized to cause severity in dengue [12] and hence, from the laboratory confirmed/presumptive patients, 227 were selected by their ethnicities (Malay, Chinese and Indians- excluding those with mixed parentage) for HLA-A and HLA-B typing. Eighteen HLA-A and 29 HLA-B alleles were detected in both the control and diseased groups. In both with and without warning signs groups, 5 HLA-A alleles (A*01, A*02, A*11, A*24 and A*33) and 5 HLA-B alleles (B*13, B*15, B*18, B*35 and B*40) were detected at frequencies higher than 5% (Figure 3). Patients without warning signs also had increased frequencies of while those with warning signs had increased B*44 and B*58. Four alleles, B*14, B*42, B*47 and B*54 were not detected in the diseased group. Stratification by ethnicity showed that Malay patients with and without warning signs had A*02, A*11, A*24, A*3, B*15, B*18, B*35 and B*40 at higher AF. Meanwhile, A*01 and B*13 was only increased in Malay without warning signs patients whom also had A*34, B*07 and B*58 at higher AF. In the Chinese patients, A*01 was not detected at all, while B*15 was detected only in without warning signs and B*35 in those with warning signs. Several other alleles were up-regulated in the Chinese patients including B*38, B*46 and B*58. While most alleles were highly expressed in the Indian groups of patients, A*33, B*13 and B*18 were only increased in patients with warning signs. Furthermore, alleles B*51, B*57 and B*58 were also highly expressed in this group of Indian patients. In this case- control retrospective study, the odds ratio was calculated in order to find HLA alleles association with dengue disease. Alleles, A*34 and B*15 were found to have positive associations with patients without warning signs regardless of ethnicity, whereas A*03 was negatively associated with this group of patients (Table 5) when compared to the healthy donors. In those with warning signs, allele B*15 had a positive association whereas A*03 had a negative association (Table 5). When patients with and without warning signs were compared, allele A*33 was positively associated with increased risk of having warning signs and having allele A*34 could possibly minimize the chances of developing warning signs (Table 5).

**Table 5:** HLA association with dengue of the study population.

Without Warning Sign vs. Controls	Control- AF (%)	Without WS-AF (%)	OR	95% CI	P value
**Total Population**					
A*03	9.5	2.4	0.239	0.054–1.055	0.044
A*34	1.1	9.8	10.16	2.108–49.00	0.001
B*15	12.6	24.4	2.231	1.152–4.323	0.02
**Chinese**					
A*24	9.4	35.7	5.37	1.352–21.33	0.023
**With Warning Sign vs. Controls**	**Control- AF (%)**	**With WS-AF (%)**	**OR**	**95% CI**	**P value**
**Total Population**					
A*03	9.5	2.7	0.264	0.119–0.584	0.001
B*15	12.6	19.6	1.689	1.025–2.781	0.045
**Malay**					
A*03	9.4	1.2	0.121	0.029–0.500	0.003
**Chinese**					
A*24	9.4	24.3	3.101	1.138–8.451	0.037
**Indian**					
B*57	6.5	25	4.833	1.398–16.72	0.016
**Without Warning Sign vs. With Warning Signs**	**Without WS- AF (%)**	**With WS-AF (%)**	**OR**	**95% CI**	**P value**
**Total Population**					
A*33	6.1	15.3	2.787	1.080–7.189	0.032
A*34	9.8	3	0.282	0.110–0.725	0.011
**Malay**					
A*34	13	4.5	0.3197	0.118–0.868	0.028

### IFN-γ T-cell Response in the Dengue Population

**Figure 4:**
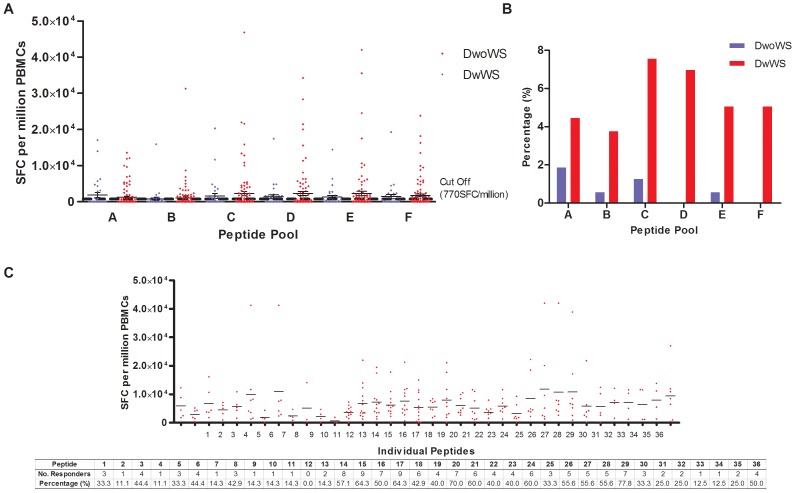
IFN-γ T cell responses in the study population. (A) SFC per million PBMCs of patients with (red) and without (blue) warning signs in response to pooled peptide. The mean cutoff values after subtraction of healthy control is at 770SFC/million PBMCs. Mean ± SEM is given for every peptide pool. (B) Number of responders towards each peptide pool for patients with (red) and without (blue) warning signs is given in percentage. (C) SFC per million PBMCs of patients in response to individual peptides. The mean cutoff values after subtraction of healthy control is not given as it varied for every peptide. The mean response for each peptide is indicated in the mean line. Table below graph shows each individual peptide, and the number of responders towards these peptides.

Presentation of antigenic peptides by the HLA molecules on APCs will activate cell-mediated immunity. Improper activation of these cells, mainly the cytotoxic T lymphocytes, has been suggested to play a role in dengue severity. Therefore, we sought to investigate the T cell activation of dengue infected patients via IFN-γ ELISpot. First, the designed peptides were pooled to evaluate the T cell responses of 156 dengue positive patients from the study population who were HLA-typed. 78.2% patients were with warning signs whereas the rest were patients from the dengue positive ND and DwoWS groups. Patients in the “without warning signs” group did not exhibit any warning signs or severe manifestations. A positive response was ascertained for when the number of SFC per million PBMCs of patients were twice as much as that of negative control wells, and above the cutoff point generated from ELISpot data of the healthy donors. Twenty-five dengue patients displayed positive T cell responses to at least one of the peptide pools and 84% were patients with warning signs. Generally, patients had higher magnitude of response in peptide pools (Pool C, D, E and F) consisting of the non-structural (NS) proteins (Figure 4A and 4B). It was also observed that patients with warning signs had larger magnitudes of responses; however no significant differences were found between patients with and without any signs in any of the pooled peptides. All 25 pooled peptide patients responded to at least one individual peptide (Figure 4C). Generally most patients tested responded to peptides designed based on the non-structural proteins where peptide 15 (NS1_187–195_ AVKDERAVH) and peptide 17 (NS2A_179–188_ PLCLSTTSQK) had the highest number of respondents. This was followed by peptides 16 (NS1_297–306_ SLRTTTVSGK), −20 (NS2B_5′–13_ NEGVMAVGL), −29 (NS4B_159–167_ VVYDAKFEK). In the non structural region, NS5 had the lowest T cell response among the patients tested. The response in the structural protein was much lower, with the capsid region peptides generating the most responders.

### Cytokine Response in the Dengue Population

Cytokines are important immunomodulators and improper T cell activation can lead to cytokine storms which are believed to result in endothelial permeability and leakage, a typical feature of dengue disease progressing into a more severe stage. Many cytokines have been implied in causing severe dengue manifestations, and in this study, 42 patients who were HLA-typed with positive/borderline T cell response and with distinct characteristics such as absence of any warning signs in the “without warning signs” group and vice versa, were included. Of the 33 cytokines studied, none showed differential expression between patients with and without warning signs. However, when compared to the controls, 12 cytokines were notably differential expressed at the different phases of illness (Table 6). IL-7 and MIF was significantly increased in patients with warning signs at all 3 phases of illness. During febrile and defervescence, the levels of CCL11, IL-8 and IL-10 was decreased considerably in patients with warning signs while cytokines CXCL10 and VCAM-1 was highly expressed. FGF-2 was only significantly decreased during febrile while ICAM-1 was increased during defervescence in those with warning signs. At convalescence, IL-5, IL-13, and IFN-γ were significantly higher in patients with warning signs. Levels of CXCL10 and VCAM-1 were also extensively increased in patients without warning signs during defervescence.

**Table 6:** Significant cytokines at different phases of illness when compared to the controls.

Cytokines	Controls	Febrile	Defervescence	Convalescence
**Inflammatory cytokines**				
IL-5	2.63±0.90	–	–	DwWS ↑↑ (9.34±1.07)
IL-10	11.32±1.89	DwWS ↓↓ (0.03±0.01)	DwWS ↓↓ (0.02±0.01)	DwoWS ↓↓ (0.01±0.00)
IL-13	8.42±1.90	–	–	DwWS ↑↑ (31.61±3.41)
IFN-γ	157.20±32.98	–	–	DwWS ↑↑ (408.60±24.87)
MIF	267.19±53.76	DwWS ↑↑ (2497.00±341.90)	DwWS ↑↑ (2201.00±354.50)	DwWS ↑↑ (5166.00±3444.00)
**Chemokines**				
IL-8	64.28±11.27	DwWS ↓↓ (37.06±9.47)	DwWS ↓↓ (27.21±2.14)	–
CCL11	184.40±16.32	DwWS ↓↓ (126.90±29.12)	DwWS ↓↓ (101.80±13.22)	–
CXCL10	1115.00±250.50	DwWS ↑↑ (172084.00±90626.00)	DwoWS ↑↑ (115716.00±87093.00)	–
			DwWS ↑↑ (41443.00±10265.00)	
**Adhesion molecules**				
ICAM-1	79313.00±5389. 00	–	DwWS ↑↑ (228076.00±25693.00)	–
VCAM-1	203603.00±23245.11	DwWS ↑↑ (460438.0±39075.0)	DwoWS ↑↑(430763.0±48866.0)	–
			DwWS ↑↑(461180.0±46475.0)	
**Growth factors**				
IL-7	8.59±1.93	DwWS ↑↑ (130.10±14.37)	DwWS ↑↑ (142.90±20.93)	DwWS ↑↑ (242.20±24.80)
FGF-2	114.00±28.240	DwWS ↓↓ (45.83±4.72)	–	–

## Discussion

Selangor Darul Ehsan, is a highly populated state of Malaysia and has the largest metropolitan area known as the Klang Valley. Thus far, this state has had the highest dengue incidence rates. Our study was conducted in 2 major government-based hospitals located in the Klang Valley situated about 50 km apart. Both the average age of the study population and the racial segregation of the study participants reflect the national age and ethnicity profile [1]. As observed in our study, the high numbers of male dengue patients have also been reflected in not only the Malaysian national dengue data but also in several neighboring countries including Singapore [20]. The most common clinical symptoms in this cohort would be vomiting, diarrhea, arthralgia and myalgia; followed by headaches, nausea and rashes typical of dengue manifestations. The most frequently observed warning signs were abdominal pain and tenderness, giddiness and mucosal bleeding tendencies. Although almost half of the patients were diagnosed with hepatitis, only 22% had hepatomegaly and liver tenderness.

Currently, the most widely used dengue diagnostic assay in hospitals is the dengue IgM ELISA [21]. Dengue NS1 detection in Malaysian hospital settings during 2010–2011 was not obligatory, therefore the lower administration of this particularly useful test in the hospital. With no pathognomonic clinical features to distinguish dengue from other febrile illnesses, laboratory diagnosis is very important. As dengue IgM can remain in the human body for 3 months [3] and may hinder the effort to detect acute dengue infections, we opted to perform other diagnostic assays independently which included a separate dengue IgM ELISA and NS1 ELISA as well as RT-PCR and HI. Overall, dengue virus RNA and/or antibodies and/or dengue NS1 antigens were detected in 62.5% of the study population. Another 10.8% of these patients were presumed to have dengue while the rest (26.7%) were found to be negative. Of concern was that (i) 37% of the non dengue classified patients were positive or presumptive for dengue infection and (ii) 23.0% who were clinical diagnosed as dengue were negative for all assays. This entails the importance of diagnosing dengue with not only clinical symptoms and parameters but in combination with suitable diagnostics assays to avoid misdiagnosis and under-diagnosis. The HI assay revealed that 60.5% of the positive dengue patients had primary infections while 38.4% had secondary infections. In contrast, when inquired about having had previous dengue infection, only 25 patients were aware of having had dengue infection in the past. This highlights the fact that there could be many silent/inapparent dengue infections continuously occurring in the country [22]; concurring with the results from a recent study in Malaysia where 9 out 10 adults were postulated to have been previously exposed to dengue infection [23].

This study cohort mostly sought medical interventions in major hospitals at day 5 of illness, however this may be because most people seek healthcare treatments from primary care clinics first [24] before being referred to major hospitals in Malaysia. Generally, we found that most patients were admitted into either the observation ward and/or dengue ward immediately upon first consultation. Most patients went into the critical defervescence phase on day 6 of illness, and were clinically managed by healthcare professional by constant monitoring of blood (4 draws/day), liver and kidney (singular draw/day) profiles. A typical feature of patients with warning signs is the increase of hematocrit levels with a concurrent decrease in platelet count. Although the hematocrit levels in this cohort more or less similar to those of the controls, the average platelet counts were well below the normal values. This indicates that thrombocytopenia in dengue patients remains an excellent marker to monitor disease progression; however it is unsuitable as early indicator for distinguishing those whom may develop warning signs [25]. Taken together, the prolonged clotting time and decrease in platelet counts observed in the dengue cohort could be due to DENV interacting with platelets and megakaryocytes via impaired thrombopoiesis or through peripheral platelet destruction [26] or nitric oxide activity in platelets [27].

Leucopenia has been reported to be common in dengue patients especially towards the critical phase; however in our study, the average of total white blood cell count remained in normal range. We observed a general dipping trend around day 4 to 8 of illness indicating leucopenia may have occurred in a number of patients. The lymphocytes count was lower in all patients except for the ND group during febrile phase of illness. These counts gradually increased as they moved into the defervescence phase, most likely signifying lymphocytosis during this period. Hepatomegaly with/without tenderness is frequently reported in dengue patients, often observed with increased liver enzymes [28], [29] where this phenomenon was also noted in our cohort. The liver enzymes ALT and AST have been indicated to serve as early markers for manifestation of severe dengue [25], however, once again with the 2009 dengue classification noteworthy differences between patients with and without warning signs were not found.

The main limitation of this study is the small number of patients in the ND and SD group which disallowed us to independently analyze each dengue classification as its own. Hence, clinically ND classified patients who were found to be dengue positive/presumptive and did not display any warning signs were included in group “without warning signs” while the SD patients whom all displayed warning signs were grouped as “with warning signs” for host genetic and immunological characterization. The MHC molecules including HLA alleles are important for the activation of T lymphocytes immune response [30], and these genetic factors have been implicated in disease susceptibility [31] including dengue [32]. A recent study in Malaysia has indicated that indeed the HLA alleles may play a role in dengue susceptibility and protection [33]. Looking at the diseased cohort as a whole, A*03 is portrayed as a candidate allele for dengue protection as demonstrated in earlier dengue studies [33], [34]. HLA-B*15, with a positive association with dengue infection, was previously found also to be susceptible in the Cuban population (38), contrarily, protective in the Venezuelan population [34]. Being one of the most polymorphic genes, HLA will have substantial differences in different ethnic groups [35], and since Malaysia is multiracial country, the study population was segregated ethnically with surprising results, where A*24 was notably associated with dengue susceptibility in the Chinese only. Allele A*24 has been previously associated with dengue susceptibility and severity in dengue patients from Vietnam [32], Jamaica [36] and Sri Lanka [37]. In Indian patients with warning signs, allele B*57 was found to be positively associated with dengue as was previously shown in the Venezuelan population [34]. HLA-A*03 which could be a candidate gene for dengue protection was only found to be associated with Malay dengue group. HLA-A*34 has not been associated with any infectious disease thus far, however, has positively associated with the dengue patients without warning signs. Further analysis revealed that this allele may be resistant against development of warning signs and with ethnicity breakdown, only to be associated in the Malay cohort. HLA-A*33, on the other hand, seems to promote disease severity or at least the development of warning signs in dengue patients as was shown before in cohorts of Thais [38] and Vietnamese [39]. Nevertheless, up scaling the number of dengue-infected individuals and in other locations would be required to assess the true potential of these associations in the Malaysian dengue patients.

Viral antigen presentation on host cells by the HLA molecules will subsequently activate T cells, and the major concern in dengue, is the presence of memory T cells which appear to be cross reactive [40] and/or inefficient in clearing virus [41]. In an endemic country like Malaysia, where most people are exposed to the virus, the majority of the population will eventually have memory T cells. Hence, viral peptides were designed based on major HLA molecules in Malaysians [33] to elucidate IFN-γ T cell response in this study population. Majority of positive responders with higher magnitudes of responses were patients with warning signs, indicating that perhaps these people may have more activated circulating CD8+ T cells [42]. Peptide pool C consisting of peptides targeting the NS1 and NS2A region had the highest number of responders indicating these regions may contain important T cell epitopes. The NS1 protein, believed to be involved in DENV RNA replication, has been correlated with dengue severity [43], [44], whereas the NS2A region is believed to necessary for processing of the NS1 protein [45]. Despite the high number responders to peptides in NS1 and NS2A areas, the highest magnitude of response seems to be peptides targeting the NS4A and NS4B regions. These NS proteins of DENV appear to be involved in the membrane localization of the viral RNA replication complex [46].

The ability of our patients to evoke cytotoxic T cell responses has prompted the study on cytokine levels in these patients, because such immune activation could trigger a cascade of events eventually causing an impromptu or overdriven cytokine storm [12], [47]. Amongst the 33 cytokines levels analyzed in our cohort, none displayed significant differences between patients with and without warning signs; however, 12 were notably different from healthy controls. IL-7, important for B and T cell development, was found to be highly expressed in patients with warning signs throughout the disease and this was also observed in a severe dengue cohort from Brazil [11]. Another cytokine that was increased in patients with warning signs is the macrophage inhibition factor where it has been correlated to dengue severity in various other studies [48], [49], [50]. This cytokine has been postulated to induce and mediate production of other pro-inflammatory cytokines [50], triggering a cascade of cytokine leading to vascular permeability seen in dengue patients. More recently, MIF induction in dengue infection was proposed to activate and stimulate endothelial cell to increase ICAM-1 expression [51]. As was observed in our study, ICAM-1 was indeed increased in patients with warning signs during defervescence, and this adhesion molecule have been indicated in endothelium damage and activation [52], [53]. VCAM-1, the other adhesion molecule, was also highly expressed in patients with warning during febrile and defervescence; however, at defervescence, even patients without warning signs had seemed to have an increase in VCAM expression. VCAM-1 not only mediates adhesion of monocytes as well as lymphocytes but is also critical for endothelial cell survival and in the previous study have been shown to be increased in dengue patients compared to other febrile illnesses [54]. Three inflammatory cytokines, IL-5, IL-13 and IFN-γ were found to upregulated in patients with warning signs during convalescence. CXCL10, was increased in patients with warning signs during febrile and defervescence and in those without warning signs during defervescence. CXCL10 is a well-known mediator of inflammatory responses where its gene has been upregulated in PBMCs of dengue patients [55]. In contrast to other studies that showed correlation of increased IL-8 and IL-10 with dengue severity [56], [57], we observed both cytokines to be down-regulated in dengue patients which may be due to low of number of actual severe dengue patients in this study. CCL11, important in migration of eosinophils and induction of CCR3-expressing endothelial cell migration, were found to be decreased in patients with warning signs during febrile and defervescence. FGF-2 is important for the establishment of stable vascular networks, in combination with PDGF and VEGF [58]. With low levels of this growth factor in patients with warning signs during the febrile phase of illness, chances of vascular permeability has increased and may have led to plasma leakage and hemorrhage observed in these patients.

The new dengue classification system has eased up diagnosis of dengue disease especially in the clinical settings [59] compared to the previous classification. However, from our findings, long term clinical markers for DF and DHF, such as thrombocytopenia and liver enzymes levels could not discriminate between patients with and without warning signs. We were also not able to determine any other clinical parameters that could discriminate between these two groups. The main shortcoming of this study would be the low number of severe dengue cases that were available and this has hindered the ability to identify markers of dengue severity. The observed association of HLA alleles with dengue could possibly provide a likely model for predicting the risk factors of acquiring dengue in the Malaysian population while T cell response study reiterates NS2A, NS4A and NS4B as well as highlights NS1 as probable immunodominant sites. Finally, despite not finding any cytokines that could discern with and without warning signs, various cytokine trends in the Malaysian cohort had been documented and warrants further investigations.

## Supporting Information

Table S1 **Designed antigenic peptides for the IFN-γ T cell ELISpot.**
(DOCX)
