# Supplementary material for: Clinical and Immunological Markers of Dengue Progression in a Study Cohort from a Hyperendemic Area in Malaysia
Source: PLoS One. 2014 Mar 19;9(3):e92021. doi: 10.1371/journal.pone.0092021 (PMC3960168; doi:10.1371/journal.pone.0092021)
Supplement: Table S1 — Designed antigenic peptides for the IFN-γ T cell ELISpot. (DOCX) [file pone.0092021.s001.docx]

**Supplementary Tables and Figures**

**Table S1: Designed antigenic peptides for the IFN-γ T cell ELISpot**

| **Pool^a^** | **Peptide ID** | **HLA** | **DENV** | **Region** | **Position** | **Sequence** |
| --- | --- | --- | --- | --- | --- | --- |
| Pool A | 1 | B*51 | DENV2 | C | 42-50 | GPLKLFMAL |
|  | 2 | A*03 | DENV3 | C | 18-26 | RVRNRVSTG |
|  | 3 | A*03 | DENV3 | C | 36-45 | GLLNGQGPMK |
|  | 4 | B*51 | DENV3 | M | 130-138 | HPGFTILAL |
|  | 5 | B*51 | DENV3 | M | 136-144 | LALFLAHYI |
|  | 6 | A*11 | DENV1 | E | 49-57 | EVTNPAVLR |
| Pool B | 7 | A*03 | DENV1 | E | 112-120 | SLITCAKFK |
|  | 8 | A*11 | DENV1 | E | 238-246 | VTFKTAHAK |
|  | 9 | A*11 | DENV1 | E | 276-284 | TTIFAGHLK |
|  | 10 | A*03 | DENV1 | E | 299-307 | YVMCTGSFK |
|  | 11 | A*11 | DENV1 | E | 352-361 | ITANPIVTDK |
|  | 12 | A*03 | DENV1 | NS1 | 187-195 | AIKDSKAVH |
| Pool C | 13 | A*03 | DENV1 | NS1 | 245-254 | KIYGGPISQH |
|  | 14 | A*11 | DENV2 | NS1 | 219-227 | EVKSCHWPK |
|  | 15 | A*03 | DENV3 | NS1 | 187-195 | AVKDERAVH |
|  | 16 | A*03 | DENV3 | NS1 | 297-306 | SLRTTTVSGK |
|  | 17 | A*03 | DENV1 | NS2A | 179-188 | PLCLSTTSQK |
|  | 18 | A*03 | DENV2 | NS2A | 91-99 | LLLRKLTSK |
| Pool D | 19 | A*03 | DENV2 | NS2A | 158-166 | AVILQNAWK |
|  | 20 | B*4001 | DENV3 | NS2B | 5'-13 | NEGVMAVGL |
|  | 21 | A*03 | DENV1 | NS3 | 64-73 | RLEPSWASVK |
|  | 22 | A*03 | DENV1 | NS3 | 240-249 | PIRYQTTAVK |
|  | 23 | A*03 | DENV2 | NS3 | 75-84 | DLISYGGGWK |
|  | 24 | A*03 | DENV2 | NS3 | 108-117 | AVQTKPGLFK |
| Pool E | 25 | A*03 | DENV2 | NS3 | 442-450 | ILAGPMPVTH |
|  | 26 | A*03 | DENV2 | NS3 | 599-608 | RIYSDPLALK |
|  | 27 | A*03 | DENV2 | NS4A | 12'-20 | RLPTFMTQK |
|  | 28 | A*03 | DENV3 | NS4A | 71-80 | FLSGRGLGK |
|  | 29 | A*11 | DENV1 | NS4B | 159-167 | VVYDAKFEK |
|  | 30 | A*03 | DENV1 | NS5 | 44-52 | GLKRGETTK |
| Pool F | 31 | A*03 | DENV1 | NS5 | 433-441 | LVHRERELHK |
|  | 32 | A*03 | DENV1 | NS5 | 575-584 | KVVRVQRPAK |
|  | 33 | A*03 | DENV1 | NS5 | 204-212 | ALVRNPLSR |
|  | 34 | A*03 | DENV1 | NS5 | 517-525 | YILRDVSKK |
|  | 35 | A*03 | DENV1 | NS5 | 649-657 | RVGRERLSR |
|  | 36 | B*4001 | DENV1 | NS5 | 891-900 | REEEEAGVL |

DENV: Dengue Virus; C: Capsid; M: Membrane; E: Envelope; NS: Non-structural; ^a^Peptides were initially pooled as stated before individual peptide ELISpot was performed.
